# Alkali Metal Salts of 10,12-Pentacosadiynoic Acid and Their Dosimetry Applications

**DOI:** 10.1021/acs.cgd.1c00031

**Published:** 2021-03-25

**Authors:** Amy V. Hall, Osama M. Musa, David K. Hood, David C. Apperley, Dmitry S. Yufit, Jonathan W. Steed

**Affiliations:** † Department of Chemistry, Durham University, Lower Mountjoy, Stockton Road, Durham DH1 3LE, U.K.; ‡ Ashland LLC, 1005 Route 202/206, Bridgewater, New Jersey 08807, United States

## Abstract

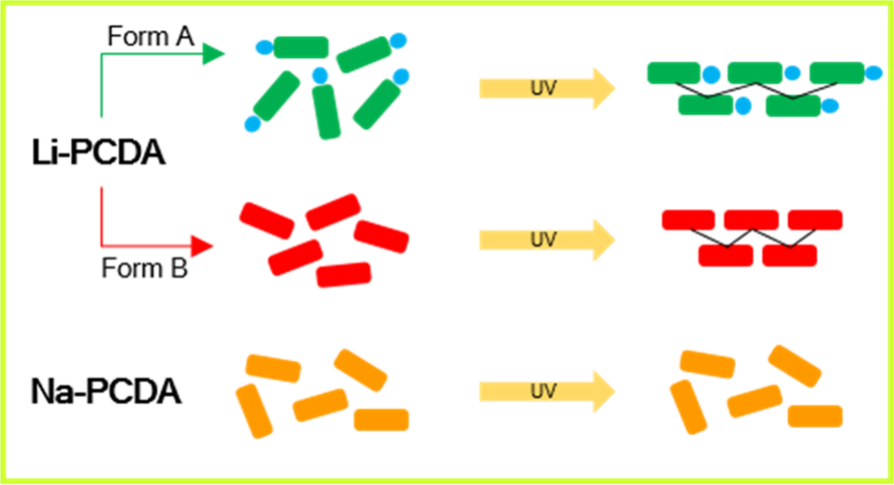

Wide-dose-range 2D radiochromic films for radiotherapy, such as GAFchromic EBT, are based on the lithium salt of 10,12-pentacosadiynoic acid (Li-PCDA) as the photosensitive component. We show that there are two solid forms of Li-PCDA—a monohydrated form A and an anhydrous form B. The form used in commercial GAFchromic films is form A due to its short needle-shaped crystals, which provide favorable coating properties. Form B provides an enhanced photoresponse compared to that of form A, but adopts a long needle crystal morphology, which is difficult to process. The two forms were characterized by powder X-ray diffraction, Fourier transform infrared spectroscopy, CP-MAS ^13^C solid-state NMR spectroscopy, and thermogravimetric analysis. In sum, these data suggest a chelating bridging bidentate coordination mode for the lithium ions. The sodium salt of PCDA (Na-PCDA) is also reported, which is an ionic cocrystal with a formula of Na^+^PCDA^–^·3PCDA. The PCDA and PCDA^–^ ligands display monodentate and bridging bidentate coordination to the sodium ion in contrast to the coordination sphere of the Li-PCDA forms. In contrast to its lithium analogues, Na-PCDA is photostable.

## Abstract 2

The monohydrated and anhydrous solid-state forms of the Li-PCDA salt photopolymerise are compared to the photostable Na-PCDA salt, which remains unchanged after UV exposure.

## Introduction

**Scheme 1:**
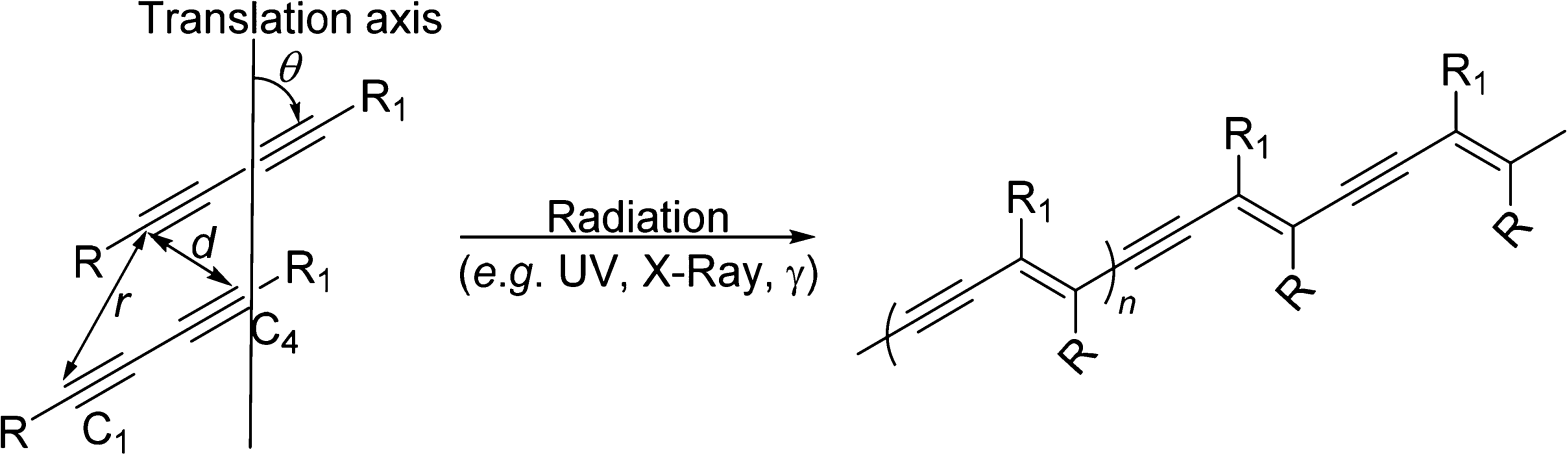
Parameters Required for the Topochemical Polymerization of Diacetylene Monomers to Result in a Polydiacetylene The C1–C4′ distance (*d*) of the monomers must be ≤3.8 Å and within the translational repeat distance (*r*) of ≤4.9 Å as well as at a tilt angle (θ) of 45°.

Radiochromic films are commercially important materials, especially in medical physics, and provide reliable and accurate dose assessments. Their mode of operation involves the polymerization of crystalline diacetylene monomers (e.g., 10,12-pentacosadiynoic acid, PCDA) to give a color change that is proportional to the radiation dose.^1,2^ It is well understood that diacetylene monomers such as PCDA can undergo a solid-state 1,4-addition polymerization reaction when exposed to heat, ultraviolet, X-ray, or γ-radiation to yield a colored polydiacetylene.^3−6^ However, the reaction will only occur under optimal topochemical conditions—the adjacent dialkyne moieties of the monomer must be separated by a distance less than or equal to the van der Waals contact distance (*d*) of 3.8 Å, have a translational period repeat spacing (*r*) of 4.9 Å or less, and have the monomers at an orientation angle (θ) to the crystal axis close to 45° (Scheme 1).^3,7,8^ The X-ray structure and topochemical parameters of PCDA were recently reported.^3^ As PCDA exhibits some photoreactivity, it has been incorporated into radiochromic films in the past (e.g., GAFchromic MD-55);^9^ however, the PCDA films are relatively insensitive and can only be used to measure doses around 5 Gy.^9,10^ More recently, sensitive self-developing films such as GAFchromicExternal Beam Therapy (EBT) have been developed by Ashland that are based on the lithium salt of PCDA (Li-PCDA)^11−18^ and span dose ranges of 0.01–40 Gy.^1,2,19^ As the more recent models of GAFchromic EBT films (especially EBT3 and EBT-XD) exhibit high spatial resolutions,^20,21^ near-tissue equivalence,^21,22^ and dose rate and energy dependence^23,24^ along with being insensitive to visible light,^25^ they are routinely employed in low-dose gradient (e.g., a quality assurance tool in intensity-modulated radiation therapy)^25−28^ and high-dose gradient settings (e.g., brachytherapy^29,30^).^18,23,31^ Therefore, continued research into photoactive ingredients with a tunable reactivity is very important to improve dosimetry technologies for different therapeutic uses. In this paper, we highlight the spectroscopic characterization of lithium PCDA salts and the crystallographic and spectroscopic characterization of a sodium PCDA salt and compare their photoreactivities, particularly in the context of the impact of the solid-state crystal form.

## Results and Discussion

### Lithium PCDA

**Figure 1:**
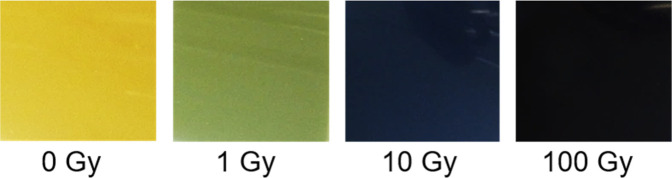
Photographs of GAFchromic EBT3 films that were irradiated with increasing doses of X-rays, ranging from 1 to 100 Gy.

**Figure 2:**
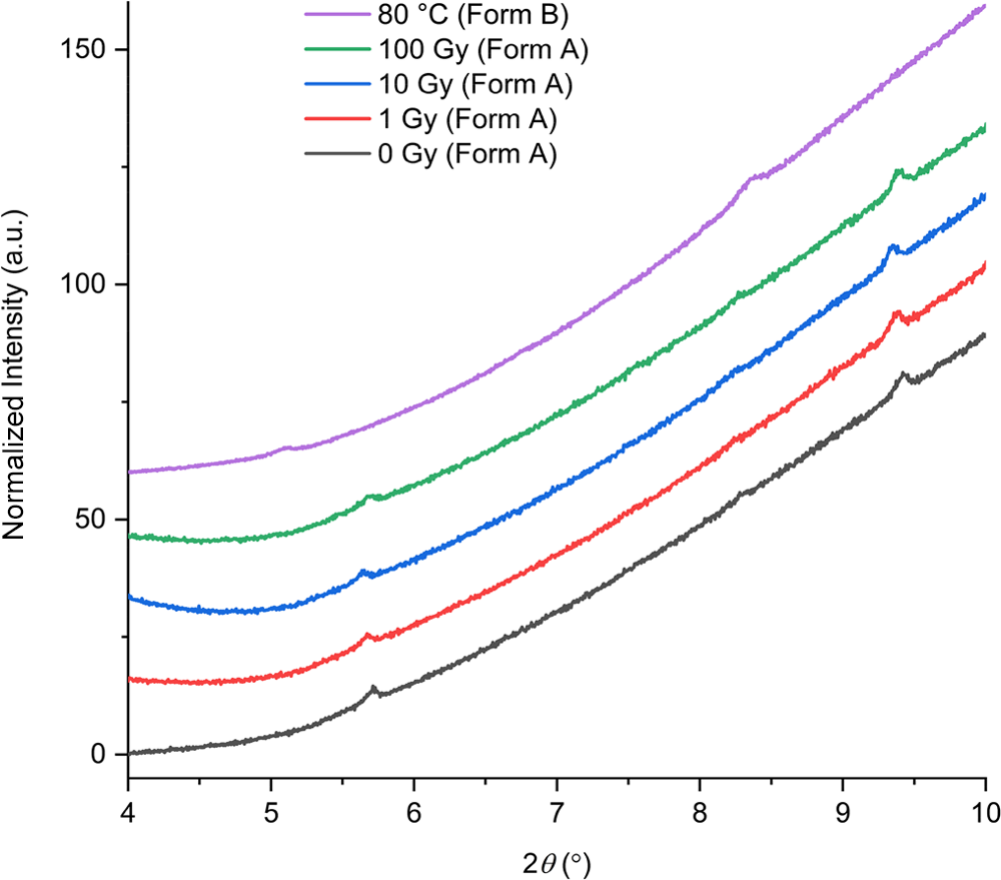
PXRD patterns of the GAFchromic EBT3 films containing form A before and after X-ray irradiation (5.7° and 9.4°) and form B after heating at 80 °C for one day (5.0° and 8.3°).

The lithium salt of PCDA is synthesized by dissolving PCDA in a basic solution (e.g., aqueous tetraethylammonium hydroxide) at elevated temperatures.^32^ When PCDA is dissolved, a 30 wt % aqueous solution of lithium hydroxide is added to form nuclei that are then ripened by Ostwald ripening to give the materials in a long needle-shaped crystal morphology. Subsequent intense sonication gives shorter needle crystals that are more suited to coating to produce GAFchromic EBT3 films. However, the shorter needles are thought to have a lower radiation sensitivity. In this work, we have examined the differences between the two crystal morphologies of Li-PCDA by powder X-ray diffraction (PXRD), which revealed that the two different morphologies represent two different crystalline forms. The short needle material, form A, is stable when kept moist, while the initially prepared long needle samples are a mixture of this form and a second crystal modification termed form B. Interestingly, the PXRD pattern of form A closely resembles the recently reported free acid PCDA^3^ with an almost identical lamellar spacing (Figure S1). The CP-MAS ^13^C solid-state NMR spectra (ssNMR) of the two forms display two peaks for form A and a single peak for form B in the carboxylate region in the range of 187–183 ppm (Figure S2), implying a lower symmetry for form A. In addition, exposing form A to heat (100 °C for 1 h or 80 °C for 1 day) or vacuum (1 day) gives pure form B (Figure S3), indicating that a phase transition occurred. Thermogravimetric analysis (TGA) experiments confirm that the transition from form A to form B corresponds to a dehydration of the monohydrated form A. TGA consistently shows a significant initial weight loss due to the moist nature of the sample, followed by a distinct weight loss step assigned to crystalline water that was calculated to be the mass of one water molecule (Figure S4). The heat-induced transformation of form A to form B was replicated in GAFchromic EBT3 films (consisting of form A). The films, based on form A, change color in proportion to the amount of radiation they are exposed to, initially starting as yellow and changing to almost black after 100 Gy of X-ray radiation (Figure 1). The PXRD patterns of the films both before and after irradiation show low-angle peaks at 5.7° and 9.4° that are indicative of the presence of form A. However, when the films are heated to 80 °C for 1 day, the Li-PCDA in the films is dehydrated and transforms to form B, as evidenced by the corresponding peaks at 5.0° and 8.3° 2θ (Figure 2).

**Figure 3:**
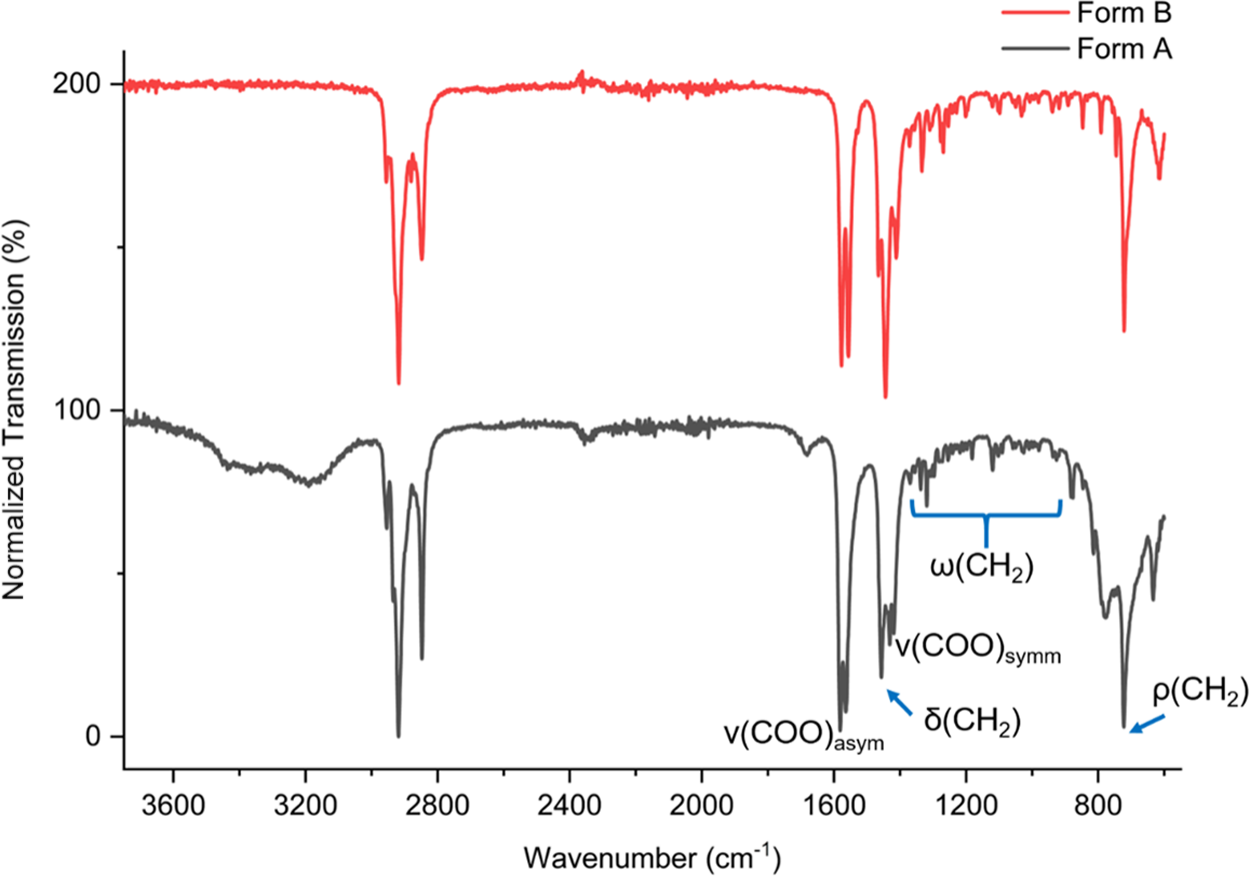
FTIR spectra of monohydrated (form A) and anhydrous (form B) Li-PCDA, with the important regions labeled. Form A also contains surface water.

**Figure 4:**
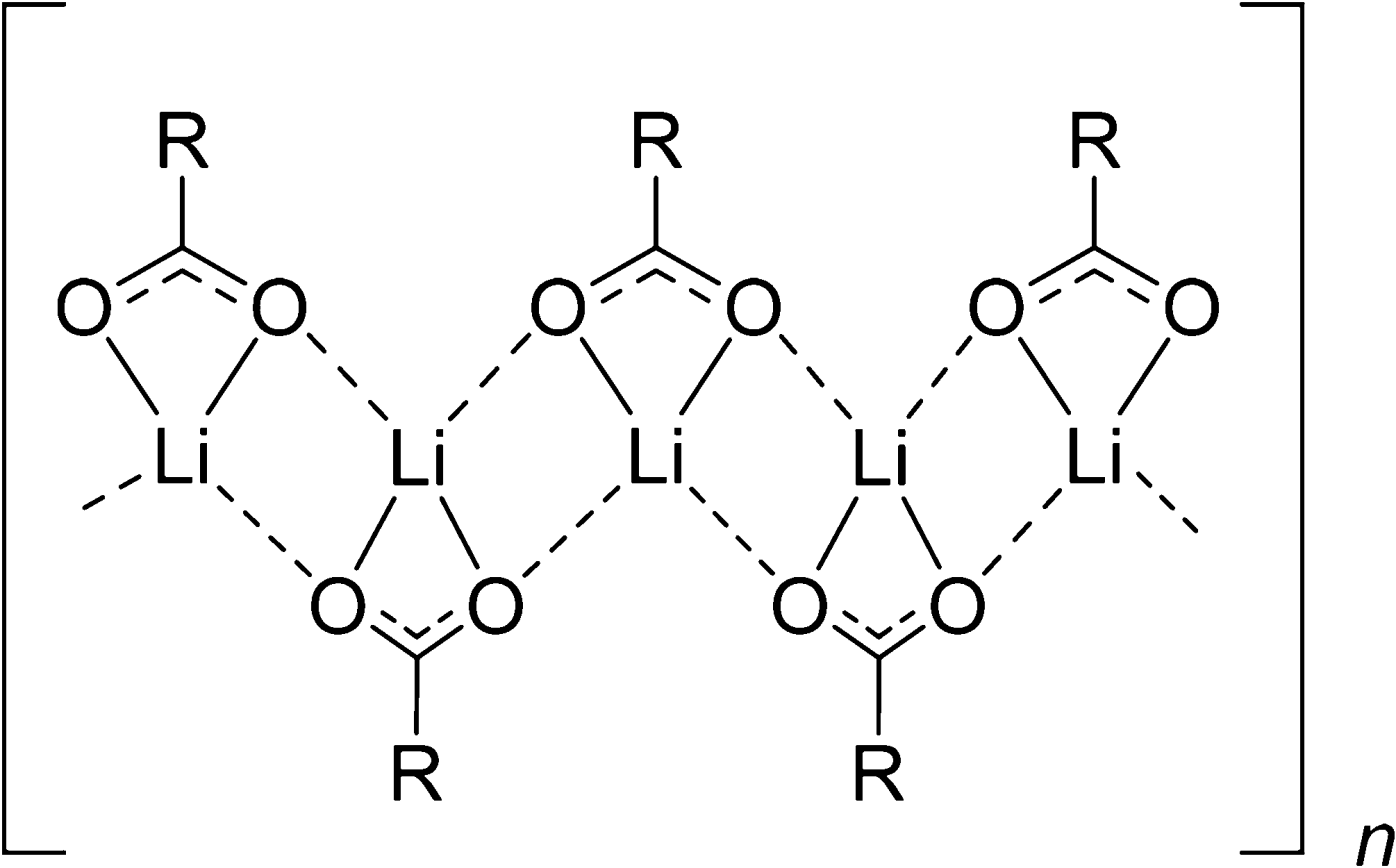
Hypothesized chelating bridging bidentate coordination environment of the lithium ion in the anhydrous form B of Li-PCDA, which was adapted from ref (34). R = C_25_H_41_.

Though neither X-ray crystal structure of monohydrated nor anhydrous Li-PCDA could be obtained as a result of the low solubility of the material, the FTIR spectra of the two Li-PCDA forms reveal insights into their structure (Figure 3). The asymmetric and symmetric carboxylate stretching bands between 1581 and 1558 cm^–1^ and 1443 and 1412 cm^–1^, respectively, are split as a result of the reduced carboxyl symmetry due to the coordination with the lithium ion.^33,34^ The carboxylate stretching bands reveal the type of coordination of the metal center, which is evident from the difference in the wavenumber of the asymmetric and symmetric stretching bands Δν = ν(COO)_asym_ – ν(COO)_symm_,^35,36^ and can be calculated from the position of the first asymmetric carboxylate stretching band minus the wavenumber of the first symmetric carboxylate stretching band. For form A, Δν is 149 cm^–1^ (1581 – 1432 cm^–1^), and that for form B Δν is 134 cm^–1^ (1577 – 1443 cm^–1^). These data are consistent with previous reports of the lithium salts of propanoic and pentanoic acids in which the Δν values are greater than the value expected for chelating bidentate coordination but lower than the range expected for bridging coordination. Therefore, a combination of chelating and bridging bidentate coordination of the carboxylate to the lithium ion is anticipated for both forms.^33,35,37^ Additionally, short-chain lithium carboxylate salts display almost identical FTIR spectra to those of Li-PCDA, with Δν values averaging 142 cm^–1^.^34,38−40^ The lithium centers of long-chain carboxylates also show asymmetric chelating bidentate bonding, which implies a tetrahedral environment of the lithium ion.^34,38,39^ This lithium environment is also expected for the two Li-PCDA forms because of the similar spectra. The proposed coordination environment of the lithium ion in form B is displayed in Figure 4, while the water molecule is likely coordinated to the lithium ion in form A. Interestingly, a progression of evenly spaced methylene wagging bands was observed in the FTIR spectrum of form B in the region of 1380–1100 cm^–1^, indicating that all methylene groups crystallized in an all-*trans* conformation similar to lithium pentanoate.^33,34^ However, form A displays additional wagging bands of a reduced intensity compared to the anhydrous material (for example, the bands at 1277 and 1269 cm^–1^), suggesting that *gauche* conformational features are present in the monohydrate structure. The Raman spectrum of form A irradiated with 100 Gy of X-ray radiation also supports the FTIR interpretation.^3^ The CH_2_ band with the greatest intensity is at approximately 722 cm^–1^ for both materials and is representative of a methylene bending vibrational mode.^34,41^ This methylene band is related to the hydrocarbon chain packing and as a result can indicate what the crystal system may be. For instance, a sharp peak without splitting (observed in form B) is indicative of triclinic or hexagonal packing,^42^ with triclinic being the most likely crystal system;^43,44^ this is in agreement with the *P*1 space group of the related Ag-docosanoate and long-chain lithium alkanoates.^38,41^ However, if the methylene band is split (as observed in form A), a monoclinic or orthorhombic crystal system is suggested, such as the *P*2_1_/*c* space group of short-chain lithium alkanoates.^38,42^

**Figure 5:**
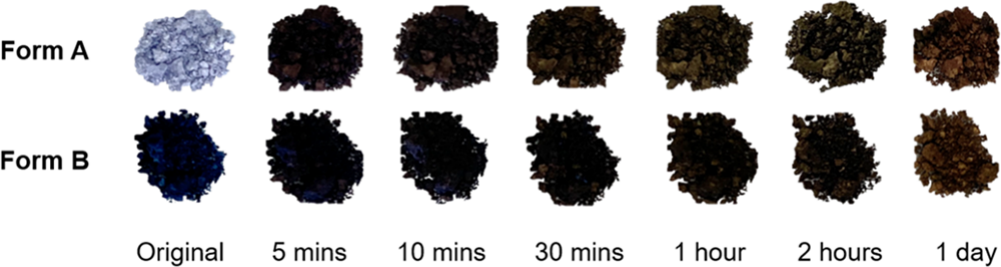
Photographs of the monohydrated (form A) and anhydrous (form B) forms of Li-PCDA powdered samples before and after different durations of UV irradiation (254 nm). As expected from the photoresponsive materials, the sample gradually darkened with prolonged radiation exposure. Note that the initial dark color of form B arises from surface coloration during the drying needed to remove form A; however, ssNMR data confirm that the bulk of the sample had not undergone appreciable photoreaction before UV irradiation.

**Figure 6:**
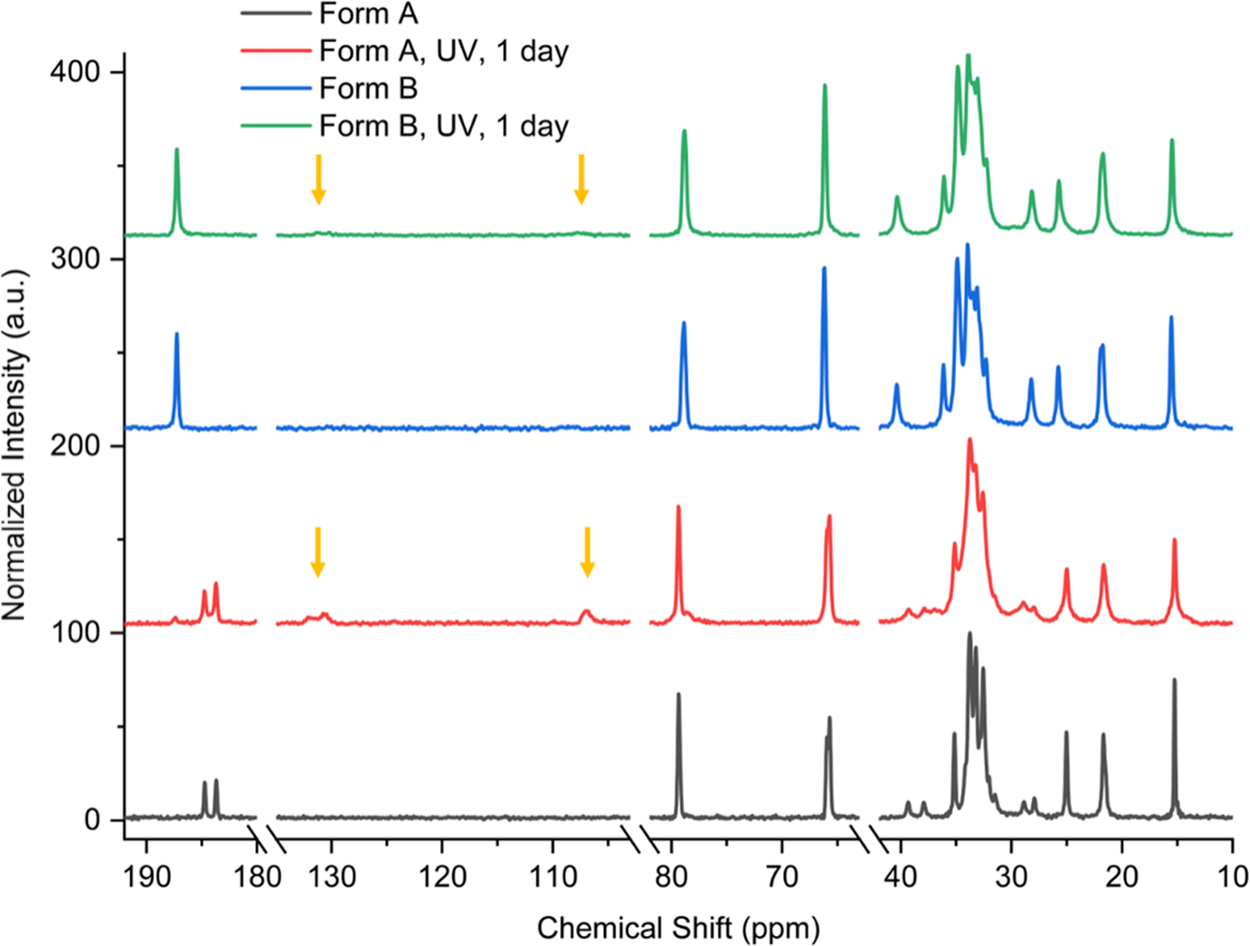
ssNMR of monohydrated (form A) and anhydrous (form B) Li-PCDA irradiated with UV (254 nm) for one day. The carboxylate region is 190–180 ppm, while the alkene peaks arising from photopolymer occur in the 130–100 ppm region. Alkyne and alkane peaks are shown in the 80–60 and 40–10 ppm regions, respectively. The *x*-axis has added breaks to compact the spectra.

While it is the monohydrated Li-PCDA (form A) material that is currently used in GAFchromic EBT3 films, it can be assumed that both form A and form B must conform to the topochemical postulate for photoreactivity, since both display radiation sensitivity. This photoreactivity may be demonstrated by placing the two powdered forms on filter paper and exposing them to UV (254 nm) radiation for varying durations. Both materials show an immediate darkening after 5 min of irradiation (Figure 5). The ssNMR spectra of form A and form B after one day of UV irradiation reveal the appearance of alkene peaks of the photopolymer as a result of the conjugated ene–yne backbone in the range of 130–100 ppm, indicating a slow conversion from the monomer to the polymer and implying a broad window of sensitivity, which is a desired property from a dosimetry perspective (Figure 6). In Ashland’s experience, a mixture of form A and form B (the presonication long needle morphology material) is more responsive to radiation in a film context; however, the short needle morphology of form A provides favorable film-coating properties. Therefore, as the different materials have altered monomer-to-polymer conversions as a function of irradiation, tuning for a range of different applications is possible.

### Sodium PCDA

**Figure 7:**
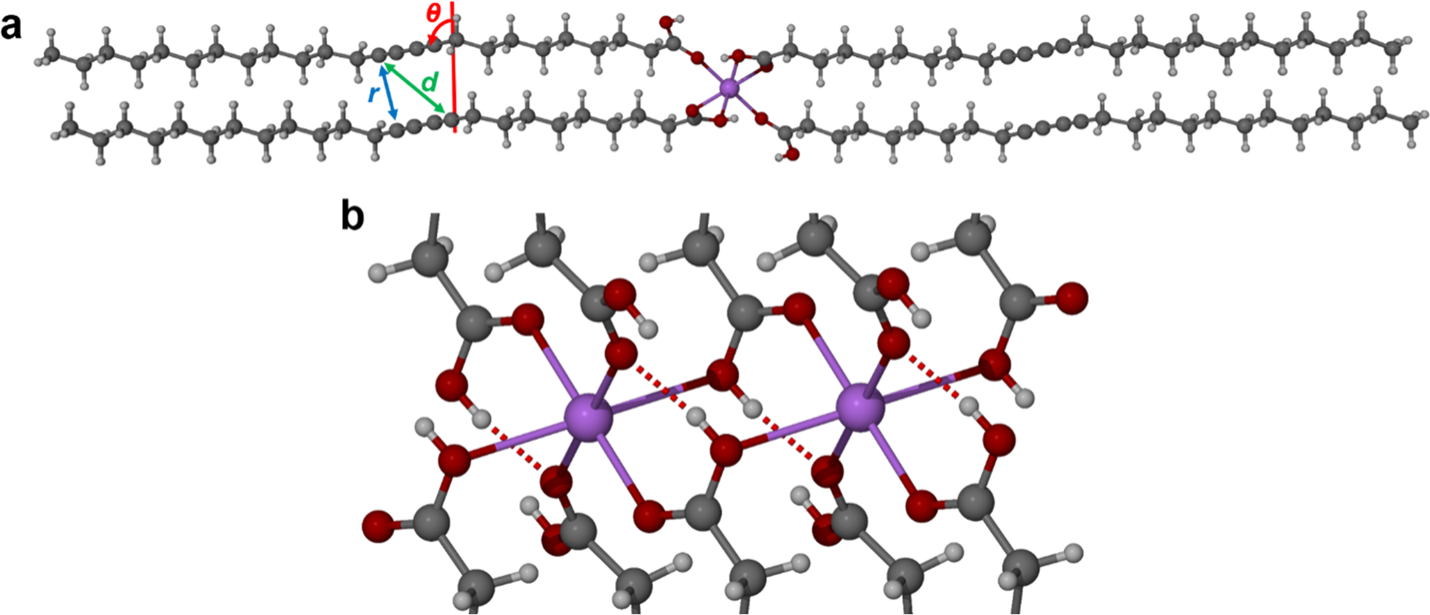
(a) The X-ray structure of Na-PCDA in the crystallographic (100) direction with the topochemical parameters added. (b) An enlarged section of the Na-PCDA head groups to show the hydrogen bonds between the PCDA acid ligands and the salt ligands (with a disordered proton).

To investigate group-one alternatives to lithium salts that may adhere to the topochemical postulate of photoreactivity, the sodium salt of PCDA (Na-PCDA) was synthesized by combining sodium hydroxide and PCDA in a ball mill for 45 min in both 1:1 and 1:3 ratios. Characterization of the 1:1 material revealed a mixture of PCDA and a Na-PCDA salt, while the 1:3 material consisted of only Na-PCDA. Serendipitous crystals of Na-PCDA were obtained from a mixture of PCDA and 3-aminopyridine by slow evaporation under ambient conditions. The resulting colorless plate crystals were analyzed at the I19 beamline at the Diamond Light Source. It was assumed that the sodium present was from contamination from the glass crystallization vial. The X-ray structure of Na-PCDA reveals a PCDA sodium salt, which is a free-acid cocrystal that crystallizes in space group *P*1, with the sodium cation bound to one deprotonated PCDA (PCDA^–^) ligand and three neutral PCDA acid ligands to give the formula Na^+^PCDA^–^·3PCDA (Figure 7a). In addition, the PCDA salt ligand has a disordered carboxylic acid proton; hence, this site represents both an anion and a neutral molecule as the proton has a site occupancy factor of 50%. The sodium cation displays a distorted octahedral geometry (average O–Na–O bond angle of 85.7°) with the sodium ion bound to the carbonyl oxygen atoms at distances of 2.3580(13) and 2.3723(11) Å, and the Na–O distance from the sodium ion to the carboxylic acid OH group of the PCDA ligands is 2.6176(11) Å (Figure 7b). Two PCDA ligands coordinate to an adjacent sodium ion by bridging bidentate (*syn*–*anti*) oxygen atoms from the carboxyl ligands to form a continuous chain, while the remaining ligands have a monodentate bonding mode that differs from the hypothesized coordination sphere of Li-PCDA. Hydrogen bonds are also present within the structure, occurring from the hydrogen atom of the carboxyl group to the neighboring carbonyl oxygen atom at an O···O distance of 2.6290(16) Å. The long PCDA aliphatic chains are in an all in the *trans*-conformation and are packed in both a bilayer arrangement and tail-to-tail, which is reflected by the long *c*-axis of the unit cell at 54.510(6) Å. The previously reported structure of sodium hydrogen dihexadecanoate is similar to that of Na-PCDA in that there are two aliphatic chains in the asymmetric unit, although the sodium ion coordination sphere involves a total of five oxygen atoms.^45^ Two of the ligands consist of hexadecenoic acid chains, while the remaining three ligands are consist of hexadecanoate chains. Similar to Na-PCDA, two oxygen atoms from adjacent hexadecanoate ligands bridge a neighboring sodium ion to give both mono- and bidentate coordination to the sodium ion.^45^ Additionally, the X-ray structure of Na-PCDA reveals that the dialkyne moieties in the structure are not within the optimal topochemical parameters for photopolymerization (*r* = 4.045(3) Å, *d* = 4.244(3) Å, and θ = 33°); therefore, the material is expected to be unreactive when exposed to radiation.

**Figure 8:**
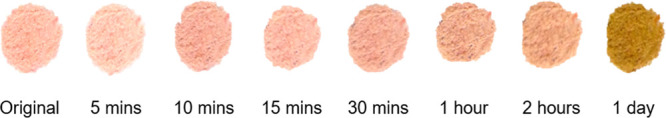
Na-PCDA irradiated for increasing durations of UV (254 nm) radiation. The powders were placed on a glass slide and exposed to radiation without any rotation of the powder so that only the surface of the powder was exposed.

As Na-PCDA does not adhere to the topochemical postulate, the powder was irradiated to investigate if the powder was useful as a dosimetry material. The powder was placed on filter paper and exposed to different durations of UV (254 nm) radiation. The powder was moved around the paper and pressed with a spatula at approximately hourly intervals to ensure the bulk of the powder was exposed to the radiation. The color of the powder remained unchanged until after one day of UV irradiation, where the powder darkened somewhat (Figure 8). Because of the lack of photoreactivity up until one day of irradiation, the powder darkening may be due to minor photodegradation. To confirm the lack of polymerization and investigate any changes in the structure upon prolonged irradiation for 7 days, Na-PCDA was analyzed by PXRD (Figure S5), FTIR (Figure S6), and ssNMR (Figure S7). Characterization of the irradiated powder provided no evidence of photopolymerization or photodegradation, which was particularly evident by the lack of alkene peaks in the region of 130–100 ppm in the ssNMR spectra.

## Conclusion

The X-ray structures of short-chain lithium salts and PCDA give insights into the structures of Li-PCDA and its monohydrated and anhydrous forms,^33^ which are further illustrated by the similarity of the lamellar spacing in the PXRD patterns.^3^ It is hypothesized that form A crystallizes in a monoclinic space group with a similar lamellar spacing to that of PCDA itself, while form B crystallizes in a triclinic space group. Both carboxylate head groups of each form have the same mixed bridged–chelating bidentate coordination to the lithium ion even though form A has a water molecule coordinated to the lithium ion. Additionally, form A can be transformed to form B in the final dosimetry film by heating. Due to the photoreactive nature of both Li-PCDA forms, they must also adhere to the topochemical postulate in a similar way to the organic salts of PCDA.^3^ Li-PCDA has been shown to produce an ordered photopolymer when irradiated (as evident by the Raman spectroscopy analysis), although PCDA combined with organic cations gives materials with enhanced photoreactivities.^3^ In contrast to Li-PCDA, the X-ray structure of Na-PCDA demonstrates a different coordination environment that involves both monodentate and bridging ligands and is not sensitive to radiation. The lack of photoreactivity in Na-PCDA reinforces the importance of the topochemical postulate to predict the photoreactivity of a system, which can only be quantitatively confirmed by the X-ray structure. Therefore, a sustained investigation into different salts of PCDA is required to continually improve and advance photoreactive materials and their dosimetry applications.

## Experimental Section

### General

Form A of Li-PCDA was supplied by Ashland LLC, and all other reagents and solvents were purchased from standard commercial sources and used without further purification. IR spectra were measured with a PerkinElmer 100 FT-IR spectrometer with a uATR attachment. Raman spectra were collected on a PerkinElmer Ramanstation 400F instrument with 5–10 accumulations of 10–60 s scans using an excitation laser with a wavelength of 785 nm. Thermogravimetric analysis thermograms were recorded using a TA Instruments Q 500 TGA analyzer. Between 1 and 5 mg of the sample was weighed into platinum pans, and dry nitrogen was used as the purge gas (flow rate of 60 mL min^–1^). Solid-state NMR spectra were recorded at 100.63 MHz using a Bruker Avance III HD spectrometer and a 4 mm magic-angle spinning probe. Spectra were obtained using cross-polarization with a 20 s recycle delay and a 7 ms contact time at an ambient probe temperature (approximately 25 °C) using a sample spin rate of 10 kHz with 400 repetitions. Spectral referencing was with respect to an external sample of neat tetramethylsilane. Single-crystal data for Na-PCDA were collected at 100.0(2) K at the I19 beamline (Dectris Pilatus 2 M pixel-array photon-counting detector, undulator, graphite monochromator, λ = 0.6889 Å) at the Diamond Light Source, Oxfordshire. The structure was solved using direct methods and refined by full-matrix least-squares on *F*^2^ for all data using the *SHELXL*(46) and OLEX2 software.^47^ All non-hydrogen atoms were refined with anisotropic displacement parameters. CH hydrogen atoms were placed in calculated positions and refined in the riding mode. H atoms attached to oxygen atoms were located on the difference map but refined as part of a rotating group (AFIX 147); disordered H atoms of the OH groups were refined with a fixed SOF equal to 0.5. X-ray powder diffraction patterns were recorded on glass slides using a Bruker AXS D8 Advance diffractometer with a Lynxeye Soller PSD detector, using Cu Kα radiation at a wavelength of 1.5406 Å. The powdered materials were placed on filter paper in a dark box and exposed to a 6 W of hand-held UV light at 254 nm.

### Synthesis of Li-PCDA and Na-PCDA

#### Lithium 10,12-Pentacosadiynoate Monohydrate (Form A)

The Li-PCDA·H_2_O supplied by Ashland contained excess surface water and was placed under vacuum at room temperature for one day before analysis (which subsequently changed the monohydrated sample to the anhydrous sample). Analysis calcd. for C_25_H_41_O_2_Li: C, 78.91; H, 10.86%. Found: C, 78.76; H, 10.76%. FTIR (cm^–1^): 3570–3040 ν(OH), 2956 ν(CH_3_)_asym_, 2934 ν(CH_2_)_asym_, 2918 ν(CH_3_)_symm_, 2847 ν(CH_2_)_symm_, 1688 δ(HOH), 1581 ν(COO)_asym_, 1564 ν(COO)_asym_, 1457 δ(CH_2_), 1432 ν(COO)_symm_, 1418 ν(COO)_symm_, 1370 ω(CH_2_), 1355 ω(CH_2_), 1338 ω(CH_2_), 1318 ω(CH_2_), 1311 ω(CH_2_), 1306 ω(CH_2_), 1297 ω(CH_2_), 1277 ω(CH_2_), 1254 ω(CH_2_), 1241 ω(CH_2_), 1231 ω(CH_2_), 1219 ω(CH_2_), 1208 ω(CH_2_), 1197 ω(CH_2_), 1182 ω(CH_2_), 1119 ω(CH_2_), 1103 ω(CH_2_), 1090 ν(C–C), 1058 ν(C–C), 1050 ν(C–C), 1030 ν(C–C), 1026 ν(C–C), 1009 ν(C–C), 999 ν(C–C), 990 ν(C–C), 979 ν(C–C), 877 ν(C–C)COO, 780 ρ(CH_2_), 722 ρ(CH_2_), 634.

#### Lithium 10,12-Pentacosadiynoate (Form B)

Li-PCDA was prepared by heating Li-PCDA·H_2_O in the oven at 100 °C for 1 h. Analysis calcd. for C_25_H_41_O_2_Li: C, 78.91; H, 10.86%. Found: C, 79.02; H, 10.78%. FTIR (cm^–1^): 2956 ν(CH_3_)_asym_, 2918 ν(CH_2_)_asym_, 2880 ν(CH_3_)_symm_, 2847 ν(CH_2_)_symm_, 1577 ν(COO)_asym_, 1558 ν(COO)_asym_, 1467 δ(CH_2_), 1443 ν(COO)_symm_, 1412 ν(COO)_symm_, 1371 ω(CH_2_), 1356 ω(CH_2_), 1333 ω(CH_2_), 1310 ω(CH_2_), 1304 ω(CH_2_), 1277 ω(CH_2_), 1269 ω(CH_2_), 1254 ω(CH_2_), 1229 ω(CH_2_), 1202 ω(CH_2_), 1195 ω(CH_2_), 1120 ω(CH_2_), 1103 ω(CH_2_), 1097 ν(C–C), 1050 ν(C–C), 1032 ν(C–C), 1026 ν(C–C), 1007 ν(C–C), 995 ν(C–C), 981 ν(C–C), 847 ν(CC)COO, 792, 747, 723 ρ(CH_2_), 617.

#### Sodium 10,12-Pentacosadiynoate

The sodium 10,12-pentacosadiynoate/10,12-pentacosadiynoic acid salt cocrystal was prepared by grinding 10,12-pentacosadiynoic acid (0.60 g, 1.59 mmol) and sodium hydroxide (0.021 g, 0.53 mmol) in a Retsch MM 200 mixer mill for 45 min at a frequency of 20 s^–1^ to yield a peach-colored powder (yield of 0.57 g, 1.15 mmol, 91%). Analysis calcd. for C_100_H_167_O_8_Na: C, 78.76; H, 10.84%. Found: C, 78.40; H, 10.81%. FTIR (cm^–1^): 2957 ν(CH_3_)_asym_, 2916 ν(CH_2_)_asym_, 2851 ν(CH_2_)_symm_, 1712 ν(C=O), 1472 δ(CH_2_), 1421, 1408, 1323 ω(CH_2_), 1287 ω(CH_2_), 1254 ω(CH_2_), 1222 ω(CH_2_), 1192 ω(CH_2_), 1100 ν(C–C), 936 νC–C(COO), 715 ρ(CH_2_). The X-ray structure of Na-PCDA came from a failed crystallization of PCDA and 3-aminopyridine in a 3:1 ratio from the slow evaporation of acetone at room temperature, which yielded colorless plate crystals. It is assumed that the presence of sodium was from a contamination in the glass vial.

#### Crystal Data

M = 1520.32 g/mol, 0.203 × 0.029 × 0.008 mm^3^, triclinic, space group *P*1 (no. 2), *a* = 5.3007(6) Å, *b* = 7.9037(9) Å, *c* = 54.510(6) Å, α = 91.674(3)°, β = 92.680(3)°, γ = 92.463(4)°, *V* = 2278.0(4) Å^3^, *Z* = 1, *D*_c_ = 1.108 g/cm^3^, μ = 0.068 mm^–1^, *F*(000) = 842.0, synchrotron radiation, λ = 0.6889 Å, *T* = 100(2) K, 2θ_max_ = 50.00°, 27970 reflections collected, 8701 unique (*R*_int_ = 0.1030). Final GooF = 1.005, *R*_1_ = 0.0998 (5841 reflections with *I* > = 2σ(*I*)), *wR*_2_ = 0.2559 (all data), 497 parameters, 0 restraints. Crystallographic data for the structure were deposited with the Cambridge Crystallographic Data Centre as supplementary publication CCDC no. 2054687.

## Supporting Information Available

Additional PXRD patterns, CP-MAS 13C solid-state NMR spectra, TGA thermograms, and FTIR spectra of Li-PCDA and Na-PCDA (PDF)

## Accession Codes

CCDC 2054687 contains the supplementary crystallographic data for this paper. These data can be obtained free of charge via www.ccdc.cam.ac.uk/data_request/cif, or by emailing data_request@ccdc.cam.ac.uk, or by contacting The Cambridge Crystallographic Data Centre, 12 Union Road, Cambridge CB2 1EZ, UK; fax: +44 1223 336033.
