# Supplementary material for: Alkali Metal Salts of 10,12-Pentacosadiynoic Acid and Their Dosimetry Applications
Source: Cryst Growth Des. 2021 Mar 25;21(4):2416–22. doi: 10.1021/acs.cgd.1c00031 (PMC8154271; doi:10.1021/acs.cgd.1c00031)
Supplement: Supplementary file 1 — cg1c00031_si_001.pdf [file cg1c00031_si_001.pdf]

Alkali Metal Salts of 10,12-Pentacosadiynoic Acid and Their Dosimetry Applications

Amy V. Hall[a], Osama M. Musa[b], David K. Hood[b], David C. Apperley[a], Dmitry S. Yufit[a], and Jonathan W. Steed[a]*

a Durham University, Department of Chemistry, Lower Mountjoy, Stockton Road, Durham, DH1 3LE, UK. E-mail: jon.steed@durham.ac.uk

b Ashland LLC, 1005 Route 202/206, Bridgewater, NJ 08807, USA

Supplementary Information

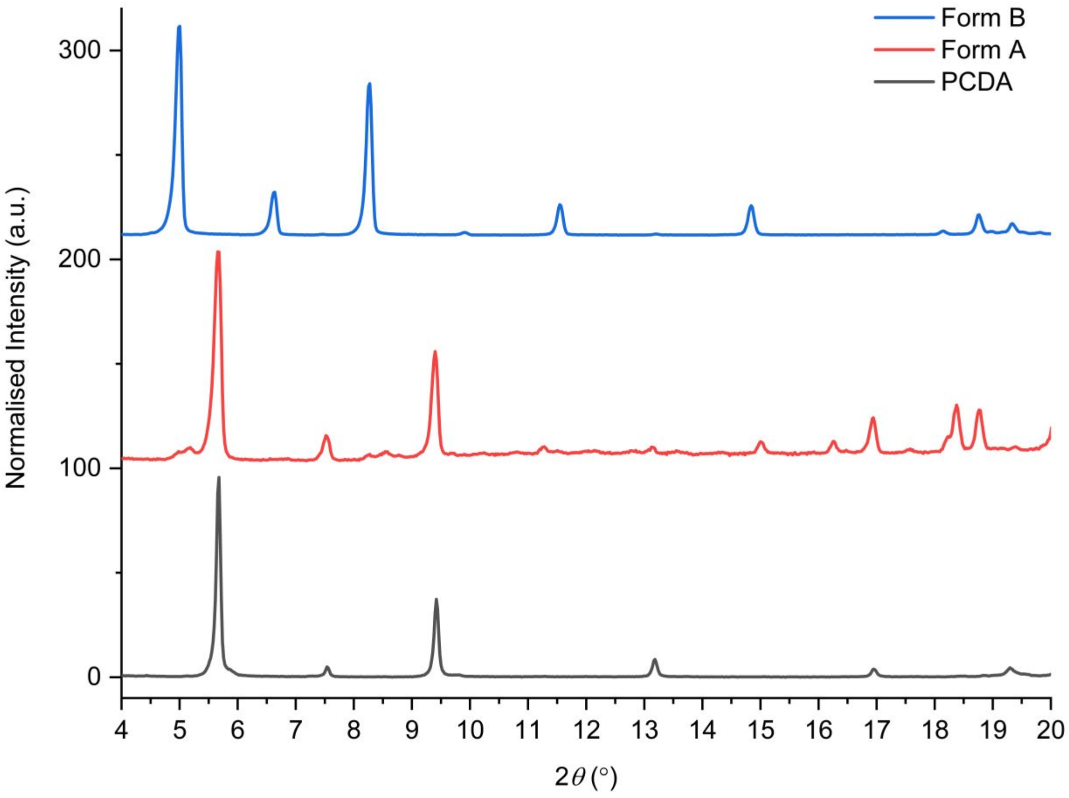

Figure S1. The PXRD patterns of monohydrated (Form A) and anhydrous (Form B) Li-PCDA, and PCDA.

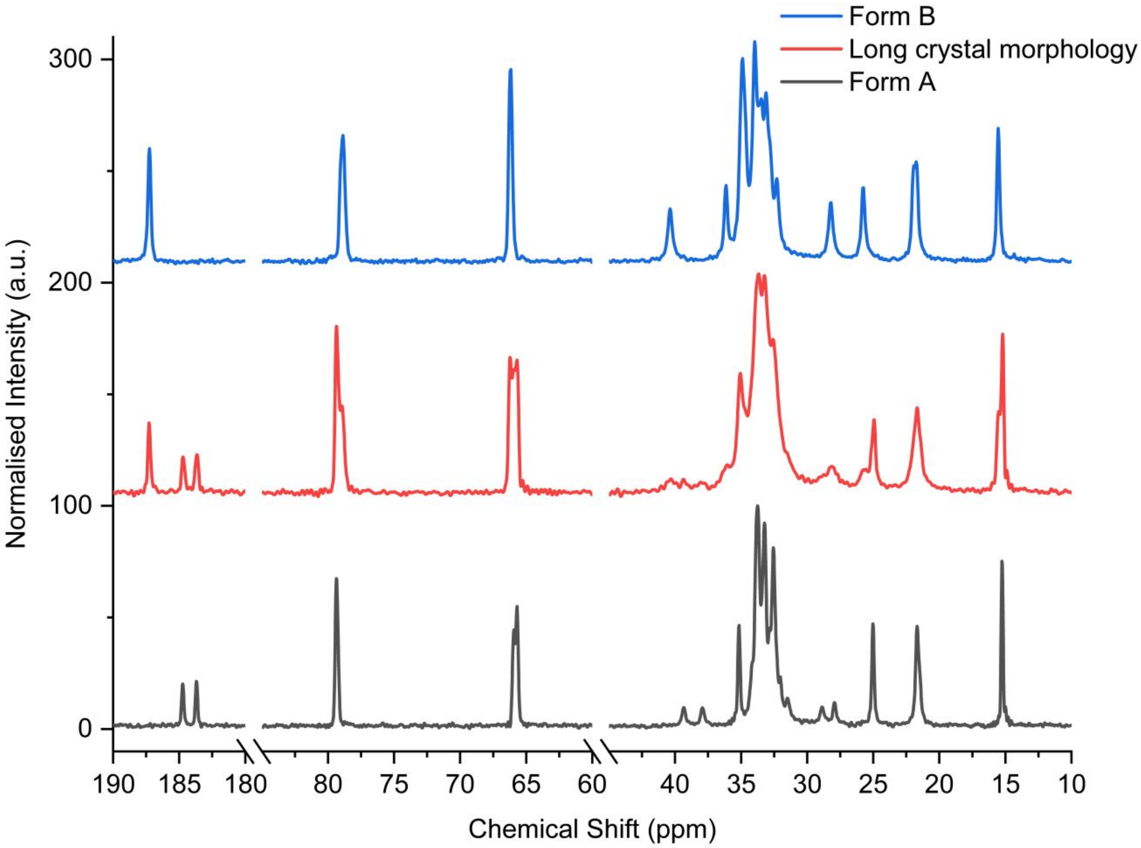

Figure S2. The ssNMR spectra of monohydrated (Form A) and anhydrous (Form B) Li-PCDA, and a mixture of the forms (long crystal morphology). The carboxylate region is 190-180 ppm while alkyne and alkane peaks are shown in the 80-60 ppm and 40-10 ppm region, respectively. Added breaks in the *x*-axis are included to compact the spectra. The carboxylate chemical shifts for anhydrous Li-PCDA structure correspond to long-chain lithium alkanoates in the literature.[1]

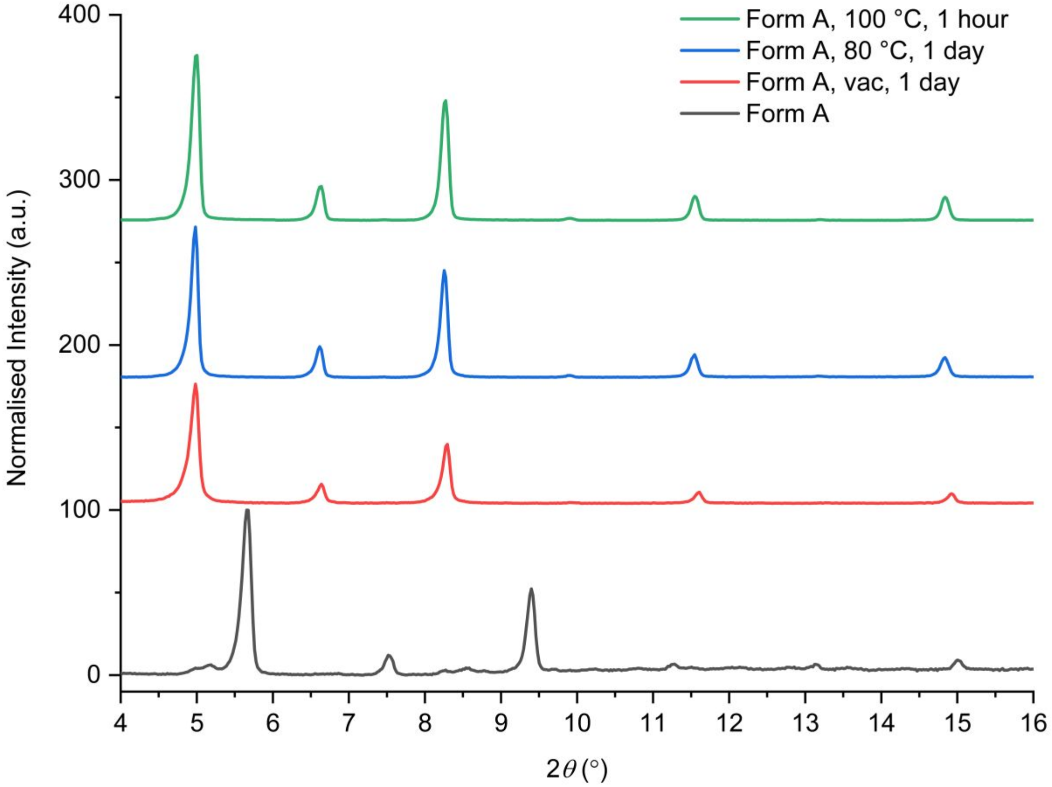

Figure S3. The PXRD patterns of monohydrated (Form A) Li-PCDA exposed to vacuum for one day, 80 °C for one day, and 100 °C for one hour, and show the conversion to anhydrous Li-PCDA (Form B).

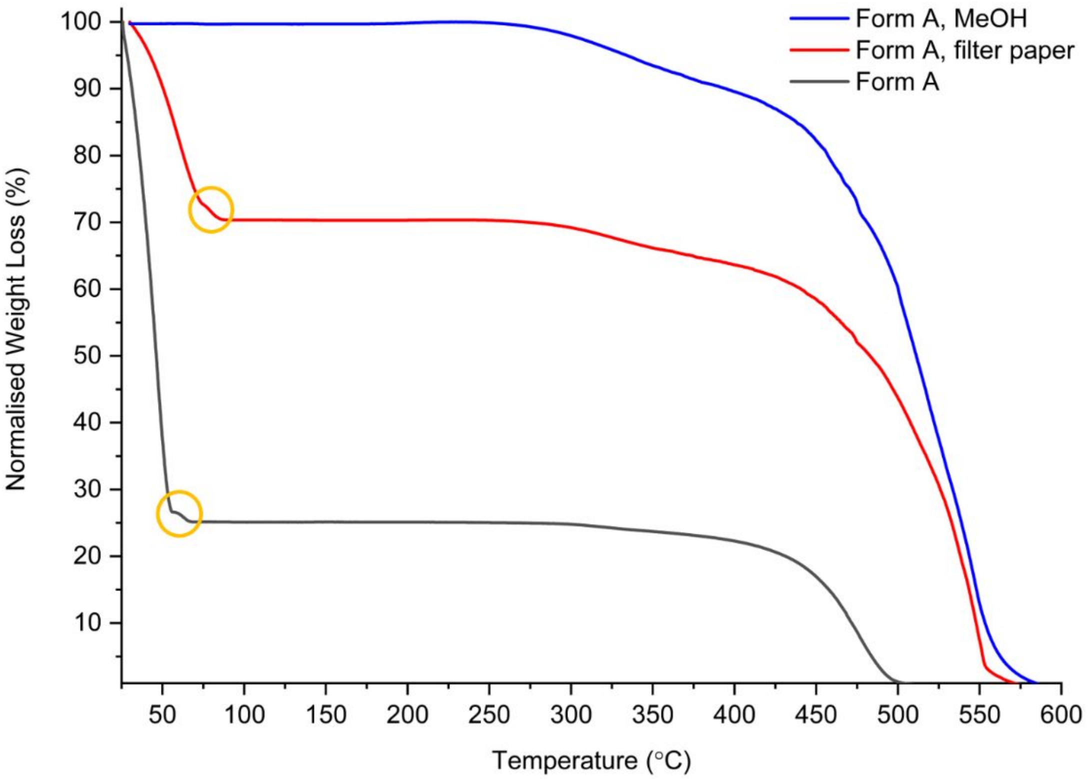

Figure S4. The TGA thermogram of moist monohydrated Li-PCDA (Form A) after partial drying by pressing on filter paper and after washing in methanol (which dehydrates the sample to give pure Li-PCDA in Form B). The yellow circle highlights the reproducible loss of crystal water which is distinct from the loss of surface moisture. This data can be used to calculate the percentage of crystalline water present in the sample. A monohydrate of Li-PCDA equates to 4.5 % water content (18 g/mol ÷ ($C_{25}H_{41}O_2Li{\cdot}H_2O$) 398.51 g/mol). Form A contains 5.6 % crystal water and after pressing on filter paper to remove the surface moisture, the crystal water content remains at 5.1 %.

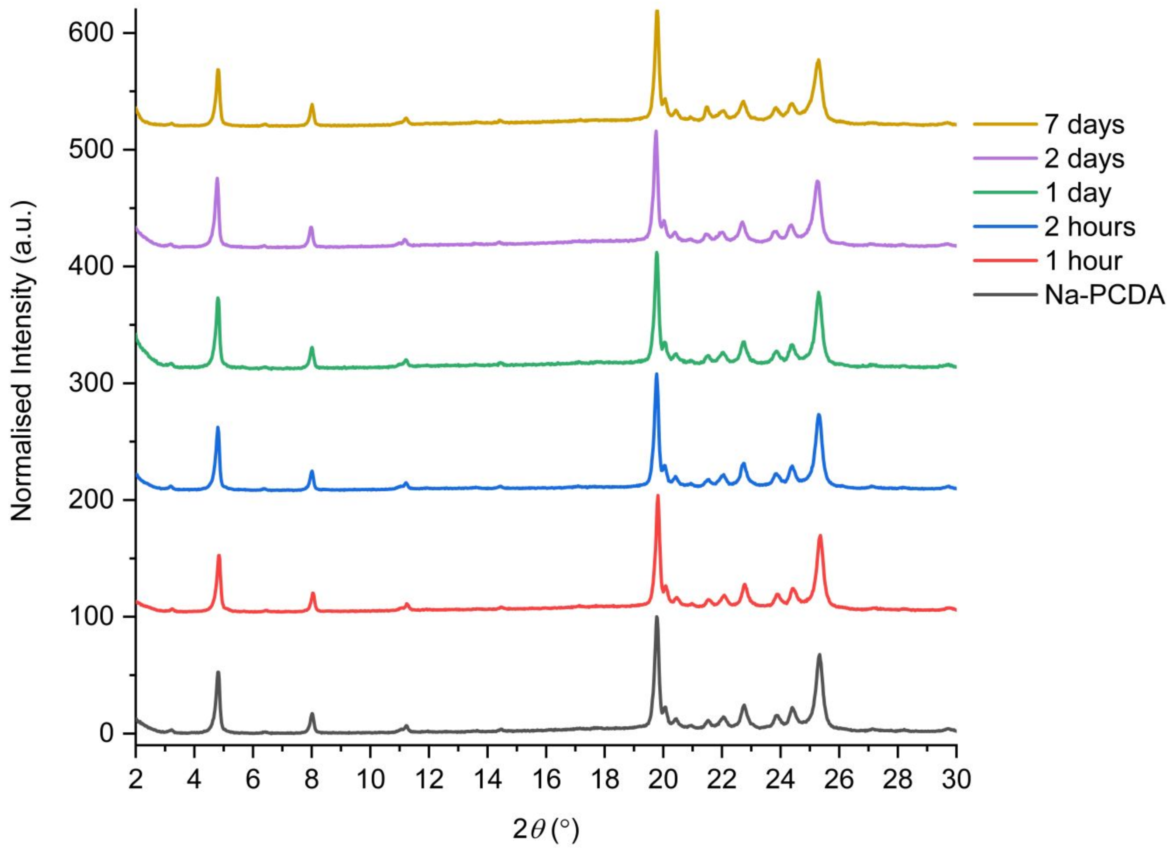

Figure S5. The PXRD patterns of Na-PCDA 1:3 irradiated with UV radiation (254 nm) for different durations.

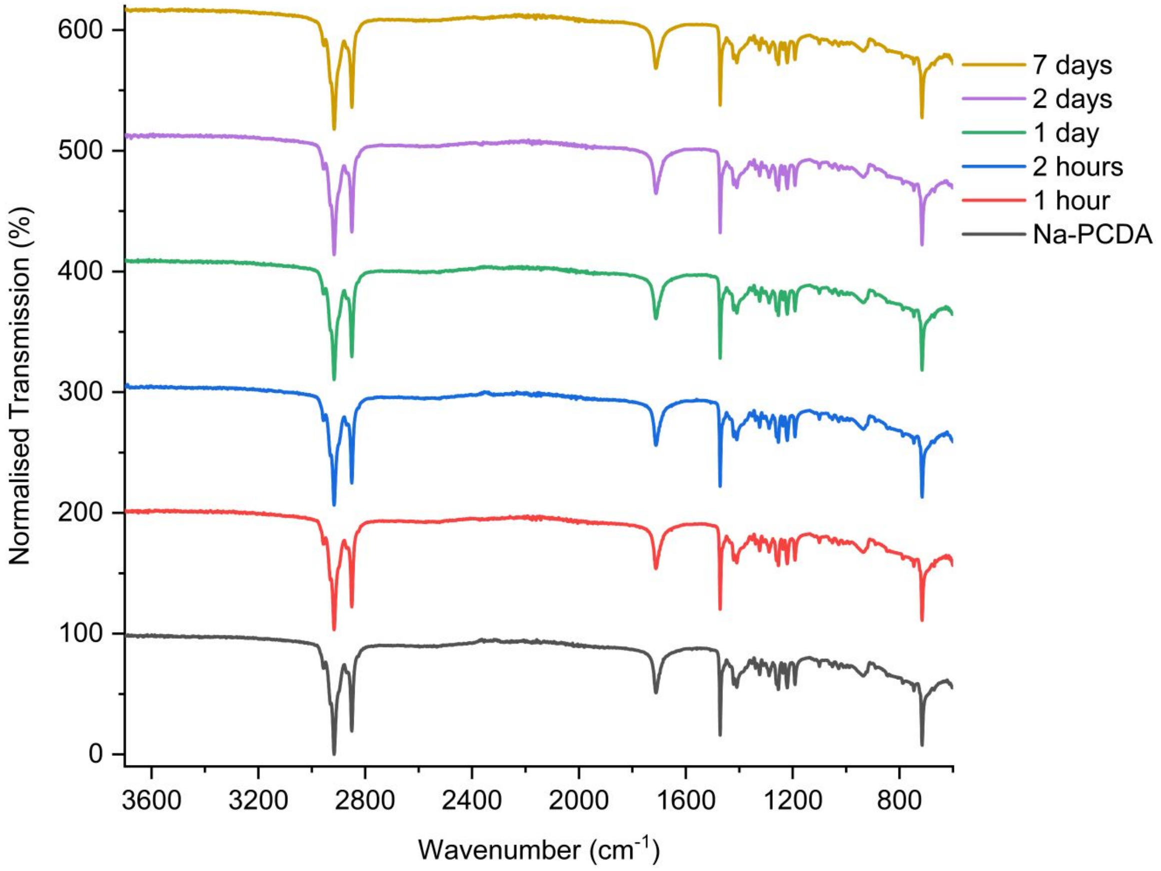

Figure S6. The FTIR spectra of Na-PCDA 1:3 irradiated with UV radiation (254 nm) for durations from one hour to seven days.

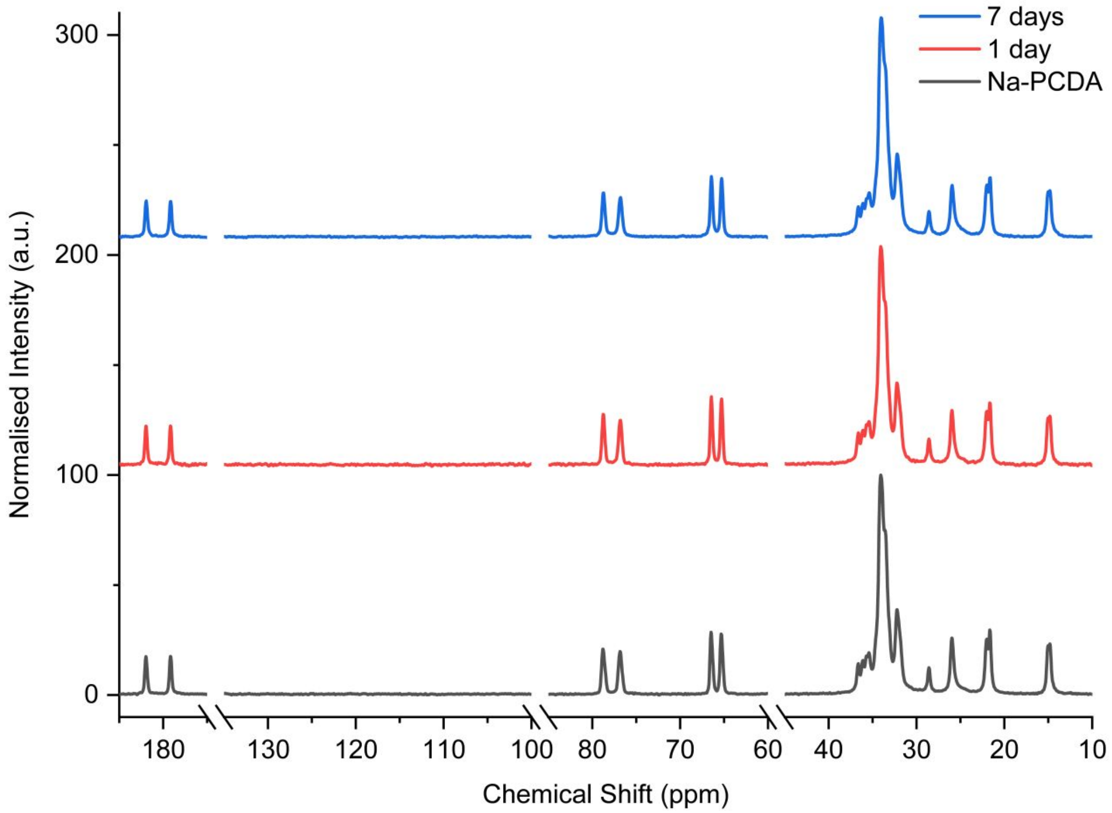

Figure S7. The ssNMR spectra of Na-PCDA exposed to UV radiation (254 nm) for one day and seven days remains unchanged. The carboxylate region is 190-180 ppm while any alkene peaks will be displayed in the 130-100 ppm region. Alkyne and alkane peaks are shown in the 80-60 ppm and 40-10 ppm region, respectively. Breaks are added in the *x*-axis to compact the spectra.

References

(1) White, N. A. S.; Ellis, H. A.; Nelson, P. N.; Maragh, P. T., Thermal and odd-even behaviour in a homologous series of lithium n-alkanoates. *J. Chem. Thermodyn.* **2011,** 43, (4), 584-590.
